# Antibacterial Activity of Orthodontic Cement Containing Quaternary Ammonium Polyethylenimine Nanoparticles Adjacent to Orthodontic Brackets

**DOI:** 10.3390/ijerph15040606

**Published:** 2018-03-27

**Authors:** Eldad Sharon, Revital Sharabi, Adi Eden, Asher Zabrovsky, Gilad Ben-Gal, Esi Sharon, Yoav Pietrokovski, Yael Houri-Haddad, Nurit Beyth

**Affiliations:** Department of Prosthodontics, Hebrew University-Hadassah, Faculty of Dental Medicine, Jerusalem 91120, Israel; DR.ELDADSHARON@gmail.com (El.S.); revital1616@gmail.com (R.S.); adieden@gmail.com (A.E.); asher.dentist@gmail.com (A.Z.); gilad.bengal@mail.huji.ac.il (G.B.-G.); esisharon@gmail.com (Es.S.); yoavpi@gmail.com (Y.P.); yaelho@ekmd.huji.ac.il (Y.H.-H.)

**Keywords:** antibacterial, nanoparticles, orthodontic brackets

## Abstract

Enamel demineralization is a common problem found in patients using orthodontic devices, such as orthodontic braces. It was found that *Streptoccocus mutans* growth increases adjacent to orthodontic devices, which may result in caries development. Incorporated antibacterial quaternary ammonium polyethylenimine (QPEI) nanoparticles were previously shown to be highly efficacious against various bacteria. Combining antibacterial materials in orthodontic cement may be advantageous to prevent bacterial outgrowth adjacent to orthodontic brackets. The aim was to evaluate the efficiency of orthodontic cement containing QPEI nanoparticles in reducing *S. mutans* and *Lactobacillus casei* outgrowth adjacent to orthodontic brackets. Orthodontic brackets were bonded to the buccal surfaces of extracted lower incisors. The antibacterial effect on *S. mutans* and *L. casei* outgrowth of Neobond bracket adhesive orthodontic cement with and without QPEI nanoparticles was compared. The antibacterial effect was evaluated using crystal violet staining and bacterial count (CFU/mL). The teeth in the experimental group, with the QPEI nanoparticles cement, showed significantly lower optical density (OD) values and CFU counts of *S. mutans* and *L. casei* than the teeth in the control group (*p* < 0.05). Based on the results, it can be concluded that orthodontic cement containing QPEI nanoparticles significantly inhibits *S. mutans* and *L. casei* growth around orthodontic brackets.

## 1. Introduction

Enamel demineralization, or white spot lesions (WSL), is a common complication found in patients using orthodontic devices, such as orthodontic braces. The white chalky appearance of the enamel is due to demineralization of the enamel underneath the surface and an increase of enamel porosity, which changes the optical characteristics of the enamel. A meta-analysis by Sundararaj at el [1], reported that the incidence of new carious lesions formed during orthodontic treatment in patients was 45.8% and the prevalence of lesions in patients undergoing orthodontic treatment was 68.4%. Factors that may increase the incidence of white spot lesions are younger age at the start of treatment, poor oral hygiene, male patients, and duration of treatment. 

The phenomenon is caused by loss of mineral from the enamel to the tooth environments. The process varies from destruction to resolution or from demineralization to remineralization, depending on the dynamic environment in the mouth. PH fluctuations, and the amount of available calcium, phosphate, and fluoride ions in the mouth, affect the diffusion out of the enamel, or back into it [2]. 

Over the years a number of preventive measures have been suggested. From the patient-side, the most important factor is meticulous oral hygiene. Other preventive measures include the use of fluoride mouthwashes and varnishes. Although these preventive measures were proved effective, patient compliance is low, especially in the adolescent population [3,4,5]. 

*Streptococcus mutans* and *Lactobacillus casei*, cariogenic bacteria, were found on orthodontic metallic brackets. It was reported that *S. mutans* growth increases adjacent to orthodontic devices and may result in caries development. Combining antibacterial materials in orthodontic cements may be advantageous to prevent *S. mutans* outgrowth adjacent to orthodontic brackets. Uysal et al. demonstrated in vivo that the antibacterial monomer-containing adhesive used was significantly more efficient than the conventional adhesive systems in reducing enamel demineralization [6]. Zinc oxide incorporated into a Fuji Ortho LC cement added antimicrobial properties to the material [7].

In a meta-analysis recently published, the efficacy of various orthodontic bonding systems antimicrobial activity was evaluated. Antimicrobial agents, such as silver nanoparticles, benzalkonium chloride, chlorhexidine, triclosan, cetylpyridinium chloride, Galla chinensis extract, acid ursolic, dimethylaminododecyl methacrylate, dimethylaminohexadecyl methacrylate, 2-methacryloyloxyethyl phosphorylcholine, 1,3,5-triacryloylhexahydro-1,3,5-triazine, zinc oxide, and titanium oxide, that were incorporated in orthodontic bonding systems showed antimicrobial activity in the agar diffusion, but when evaluated against biofilm were found to be ineffective [8].

Another method to achieve anti-bacterial effect is based on the use of antimicrobial particles that are incorporated in the material and are not released. Therefore, there is no risk of toxicity from elements that are released over time and may penetrate the tissue. These are nanoparticles based on quaternary ammonium. Incorporated antibacterial quaternary ammonium polyethylenimine (QPEI) nanoparticles were previously shown to be highly efficacious against *S. mutans*. The mechanism of action of these nanoparticles is the destruction of the bacterial cell wall [9]. 

We hypothesized that orthodontic cements combining QPEI nanoparticles will adapt antibacterial activity against *S. mutans* and *L. casei*. The purpose of the study was to evaluate the efficiency of an orthodontic cement containing QPEI nanoparticles in reducing *S. mutans* and *L. casei* outgrowth adjacent to orthodontic brackets. 

## 2. Materials and methods 

### 2.1. Preparation of Test Materials

Dry polyethyleneimine (PEI) was obtained by freeze-drying from 50% aqueous solution (Sigma Aldrich, Rehovot, Israel). 1,5-diiodopentane, 1-iodooctane, methyl iodide, sodium bicarbonate, and N-lauryl-sarcosine (NLS) (all from Sigma Aldrich, Rehovot, Israel) were used without further purification. Quaternary ammonium polyethylenimine (QPEI) nanoparticle synthesis was performed as described previously [9]. Briefly, nano-sized particles were prepared by dissolving PEI in ethanol that was reacted with 1,5-diiodopentane under reflux for 24 h. N-alkylation was conducted using 1-iodooctane. Alkylation was carried out under reflux for 48 h followed by 24 h neutralization with sodium bicarbonate. Then N-methylation, using methyl iodide, was conducted at 42 °C for 48 h followed by 24 h neutralization with sodium bicarbonate. The supernatant obtained was decanted and precipitated in double-distilled water (DDW), washed with hexane and DDW, and then freeze-dried. The average yield was ≥85% (mol/mol). Then the particles were washed with a 2% solution of surfactant (NLS). Prepared QPEI nanoparticles (20 g) were placed in a Buchner funnel using a paper filter and a vacuum source. A volume of 200 mL of NLS solution was passed through the nanoparticles under vacuum conditions. Nanoparticles were added to a commercially-available bonding material (Neobond orthodontic adhesive system, Dentsply, York, Pa, for details see Table 1), at a concentration of 1% *wt*/*wt* and mixed using a mortar and pestle, under a yellow light, until a homogeneous mass was achieved.

### 2.2. Bacterial Strains and Growth Conditions

*S. mutans* (ATCC #700610) and *L. casei* (ATCC #334) served as test organisms. *S. mutans* bacteria were cultured for 24 h at 37 °C in brain heart infusion broth (BHI) (BBL, Becton, Dickinson, and Company, Franklin Lakes, NJ, USA) supplemented with 4% sucrose (BioLab Lmt., Ltd., Jerusalem, Israel). *L. casei* were cultured at 37 °C in BHI broth for 24 h. The suspensions were adjusted to an optical density (OD) of 1 at 650 nm.

### 2.3. Teeth Preparation

**Figure 1:**
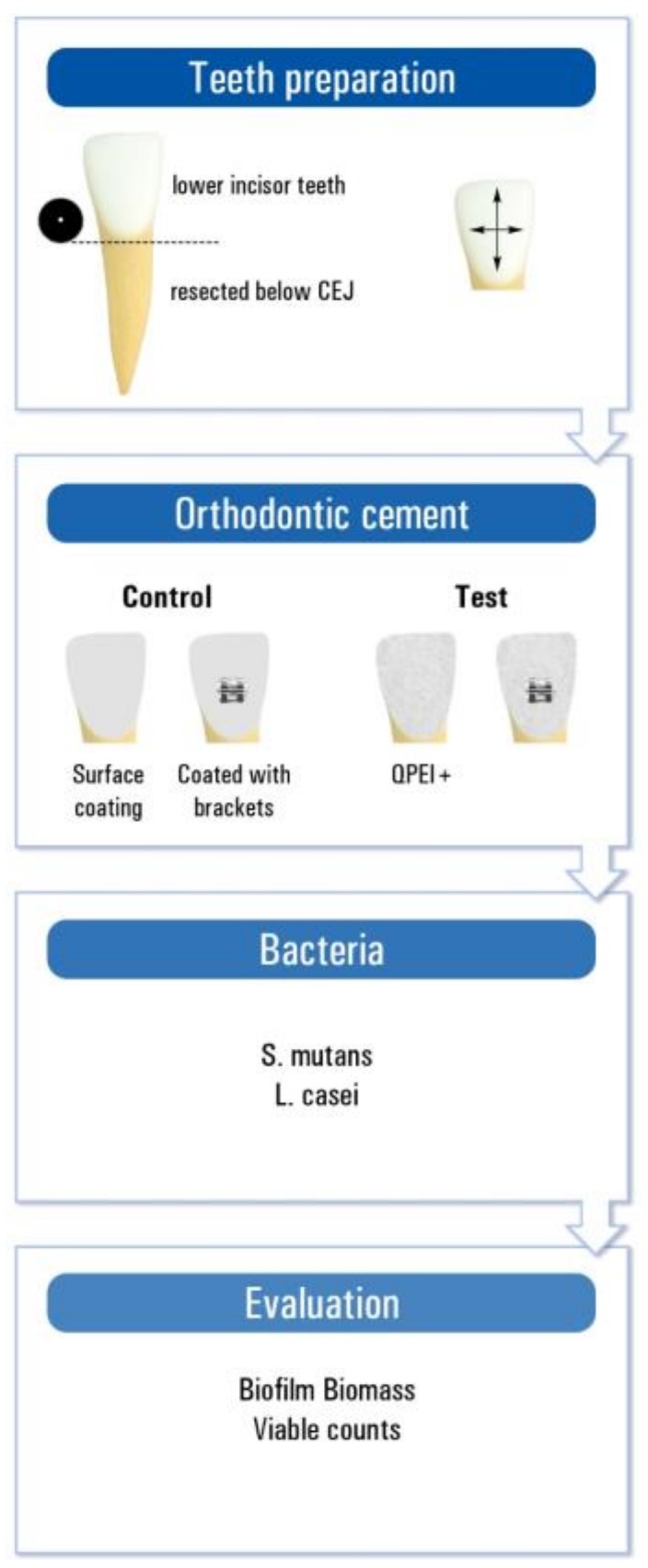
Schematic presentation of the experimental setup.

Human lower incisor teeth extracted for periodontal reasons, with no previous endodontic treatment, caries, coronal restorations, signs of resorption, or cracks, were used for this study. The teeth were kept in double-distilled water (DDW), extrinsic debris was removed, and the teeth were sterilized in an autoclave. The crown of each tooth was resected horizontally below the cemento-enamel junction. The crowns were etched with 37% phosphoric acid and adhesive and brackets were applied and then polymerized according to the manufacturers’ instructions. Test groups were coated with Neobond incorporating 1% QPEI nanoparticles (Figure 1). 

### 2.4. Bacterial Outgrowth Evaluation

Each prepared crown was inserted into an Eppendorf test tube containing 2 mL of phosphate buffered saline (PBS) and incubated for 48 h at 37 °C. The PBS was replaced with fresh PBS every 48 h for a week. After which, the samples were subjected to bacterial challenge.

One group of coated crowns served to evaluate bacterial outgrowth of simultaneously added *S. mutans* and *L. casei* (*n* = 10). An addition group served to evaluate the presence of bonded brackets on *S. mutans* outgrowth (*n* = 10). Control groups included similarly prepared crowns with non-modified Neobond (*n* = 10 each group).

A volume of 2.5 mL of the prepared suspension of *S. mutans* and a volume of 2.5 mL of the prepared suspension of *L. casei* were placed in a new test tube and the tested crown was inserted in the tube, which was then incubated for 48 h at 37 °C. The crowns with the bonded brackets were inserted in test tubes with 5 mL of the prepared suspension of *S. mutans* and then incubated for 48 h at 37 °C. The antibacterial effect was evaluated using crystal violet (CV) staining measuring the bacterial mass and bacterial count (CFU/mL) evaluating viable bacterial counts.

Following 48 h of incubation with bacteria, the crowns were transferred into a new test tube with 4 mL of PBS. The PBS was collected and replaced with a fresh one every 30 s three consecutive times. The PBS from the last collection was seeded onto a blood agar plate for viable count evaluation CFU/mL.

### 2.5. Crystal Violet Staining

For CV staining 24 well plates were used. One crown was placed in each well and 1 mL of 100% methanol was added into each well for 20 min. The methanol was then collected without touching the teeth or the well’s walls. One milliliter of 0.5% CV solution was added into each well for 20 min, and then the crown was transferred into a new well. The crowns were then washed gently three times with DDW, and 1 mL of 100% ethanol was added to dissolve the stained dye, while vortexed with a pipettor for about 30 s. Two-hundred microliters of the stained ethanol was collected from the wells into a 96-well plate, and the optical density (OD) of the samples was determined by end-point measurement using the UV–VIS spectrophotometer at a wavelength of 538 nm.

### 2.6. Statistical Analysis

Descriptive statistics was used to display the results. In order to test the significance of the difference between experimental and control groups in amount and vitality of the bacteria, paired two-sample *t*-test for means was used. The significance level was set to be 0.05.

## 3. Results

### 3.1. The Effect of QPEI on Biofilm Mass

**Figure 2:**
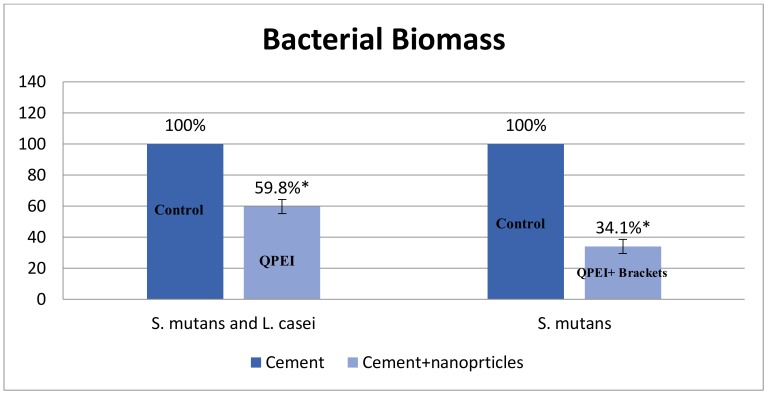
Neobond incorporating 1% QPEI (quaternary ammonium polyethylenimine) reduces bacterial biomass; * Statistically significant difference (*p* < 0.05).

The antibacterial effect of QPEI was evaluated using crystal violet (CV) staining measuring the bacterial mass. The biomass of *S*. *mutans* bacteria grown on the teeth in the presence of QPEI nanoparticles was about 34% compared to the control group, and for *S. mutans* and *L. casei* biofilm the biomass was about 60% compared to the non-treated compared group (Table 2). This reduction in biofilm mass on the surface of the tested teeth was depicted for *S. mutans* monospecies biofilm (>60% reduction) and for *S. mutans* and *L. casei* biofilm (>40% reduction) as shown in Figure 2. An inhibition of biofilm formation in the presence of QPEI nanoparticles incorporated into Neobond orthodontic adhesive system was evident in all tested samples. The differences were found to be statistically significant (*p* < 0.05). 

### 3.2. The Effect of QPEI on Bacterial Growth

**Figure 3:**
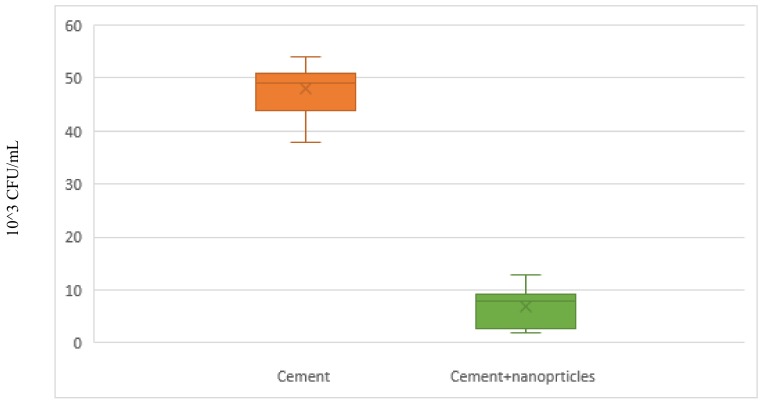
Neobond incorporating 1% QPEI reduces bacterial viable counts of *S. mutans* adjacent to bonded brackets.

The antibacterial effect of QPEI on viable bacterial growth was evaluated using bacterial count (CFU/mL). The viable cell count around the cemented non treated brackets was about 50 × 10^3^ CFU/mL. However, the bacterial viable count around the cemented QPEI treated brackets was significantly reduced (Figure 3). 

This significant reduction in viable cell counts was observed in all QPEI treated samples. An average of ninety percent reduction (*p* < 0.05) in the *S. mutans* counts was seen in all tested specimens, including QPEI nanoparticles.

## 4. Discussion

Oral hygiene during orthodontic treatment is difficult to achieve. Cariogenic bacteria around orthodontic brackets can cause enamel demineralization and caries development. In this study we modified a commercially-available orthodontic bonding material by incorporating 1% QPEI nanoparticles. The incorporation of 1% QPEI nanoparticles to the bonding agent reduced bacterial biomass by more than 40% for both monospesies *S. mutans* biofilm and the duo-species biofilm of *S. mutans* and *L. casei*. The modified bonding agent also reduced the bacterial viable counts of *S. mutans* adjacent to bonded brackets in approximately 90% showing that the study’s hypothesis can be accepted. 

The issue of enamel demineralization adjacent to orthodontic brackets led to research and studies seeking a solution for the problem. One way of dealing with the problem is adding various antibacterial materials and components to the adhesive system of the brackets, such as antibacterial monomers [6,10,11,12], silver [13], titanium [14], chlorhexidine [15], triclosan [16], and zinc oxide [7]. Apart from showing effective antibacterial activity the modified adhesive must display other features- biocompatibility, favorable physical properties (tensile and shear bond strength), and preservation of the mechanical properties of the adhesive system. 

Disadvantages of gradually released materials are deterioration of the base material demonstrating compromised mechanical properties of the adhesive, inconsistent dosage, short term effectiveness, and possible toxicity.

Quaternary ammonium particles, being polymers, are integrated into the material resin matrix. Consequently these form of antibacterial agents is more chemically stable, therefore, less toxic to the neighboring tissues, gaining the advantage of long-lasting stability.

This study showed that orthodontic cement containing 1% QPEI nanoparticles reduced and inhibited bacterial growth of cariogenic bacteria in the oral biofilm, in a realistic model as opposed to tests examining effects of antibacterial agents on the same bacteria in suspension, where they are more vulnerable.

It is recognized that the formation of a white spot lesion is preceded by biofilm adhesion. The ability to adhere to a given surface depends largely on the surface properties such as the surface topography and physiochemical characteristics. Specifically, cariogenic bacteria, such as *Streptococci mutans*, form tenacious biofilms on the tooth surface, rapidly produce lactic acid, and cause dental decay. Unfortunately, when new therapeutic approaches are suggested ethical concerns limit the ability to perform in vivo research protocols. In the present study we used human extracted teeth and bonded brackets on their surfaces. This enabled a simulation of the surface characteristics on which the biofilm attaches. Since the experimental setup was performed ex vivo we were able to control the variables and determine the effectiveness of QPEI incorporation on the tested bacteria in biofilm. 

Needless to say, in vivo testing is superior over in vitro testing allowing an observation of the large scale effects of all variables in a living subject. Thus, although this study shows positive results ex vivo of QPEI incorporation, further investigation in vivo should be performed. The ex vivo conditions in the present study enabled controlled conditions allowing careful quantification of the antibacterial effect. Unfortunately, this setup alters the “natural” conditions.

The effectiveness of the QPEI nanoparticles was assessed by evaluation of the relative reduction in viable bacterial counts in the biofilm. Viable count is a well-established means to determine bacterial viability. Unfortunately, when biofilm viability is evaluated errors can arise, the reason being bacterial aggregation due to the matrix. For this reason crystal violet staining technique was used here to measure biofilm mass. The CV staining technique has a wide range of applicability and has been developed for high-throughput studies. It is considered a suitable quantitative means for biofilm formation, since it stains the extracellular matrix polymers, the live bacterial cells, and the dead bacterial cells. These two methods, i.e., viable count assay and CV staining, complement each other, allowing a screening opportunity when evaluating an anti-biofilm approach.

## 5. Conclusions

QPEI nanoparticles were previously shown to have several advantages, including potent, broad-spectrum, and long-lasting rapid antibacterial effects. Combining QPEI nanoparticles in orthodontic cement appears to offer several advantages, as well, including significantly lowered viable bacterial counts of *S. mutans* and *L. casei*, as well as bacterial biomass around orthodontic brackets. Therefore, this method may be advantageous in preventing caries development adjacent to orthodontic brackets devices.

## Figures and Tables

**Table 1:** Neobond orthodontic adhesive system.

	Resin	Solids
Neobond Adhesive	Urethane diacrylate Triethyleneglycol dimethacrylate 2-hydroxyethyl methacrylate Photoinitiators Accelerators	Barium aluminosilicate glass Fumed silica
Neobond Primer	Urethane diacrylate oligomer Polymerization accelerators Triethyleneglycol dimethacrylate 2-hydroxyethyl methacrylate Photoinitiators	N/A

**Table 2:** Percentage of decrease in biomass for the test groups: QPEI incorporated vs. control and QPEI incorporated + bracket vs. control.

No.	*S. mutans*		*S. mutans* and *L. Casei*	
	QPEI Incorperated	Control—No QPEI	QPEI Incorperated + Bracket	Control No QPEI + Brackets
1	62.01%	100%	40.47%	100%
2	57.06%	100%	41.50%	100%
3	63.31%	100%	27.45%	100%
4	54.19%	100%	32.75%	100%
5	53.22%	100%	35.38%	100%
6	63.72%	100%	30%	100%
7	55.68%	100%	36.84%	100%
8	64.51%	100%	32.07%	100%
9	59.91%	100%	29.16%	100%
10	64.51%	100%	35.38%	100%
Average	34.10% *	100%	59.81% *	100%
SD	4.45	0	4.2	0

* Differences between groups were found statistically significant (*p* < 0.05).
